# Extracellular Vesicles in Renal Cell Carcinoma: Multifaceted Roles and Potential Applications Identified by Experimental and Computational Methods

**DOI:** 10.3389/fonc.2020.00724

**Published:** 2020-05-07

**Authors:** Zhiyuan Qin, Qingwen Xu, Haihong Hu, Lushan Yu, Su Zeng

**Affiliations:** College of Pharmaceutical Sciences, Institute of Drug Metabolism and Pharmaceutical Analysis, Zhejiang University, Hangzhou, China

**Keywords:** renal cell carcinoma, extracellular vesicles, exosomes, biomarkers, drug targets, drug vehicles, artificial intelligence, machine learning

## Abstract

Renal cell carcinoma (RCC) is the most common type of kidney cancer. Increasingly evidences indicate that extracellular vesicles (EVs) orchestrate multiple processes in tumorigenesis, metastasis, immune evasion, and drug response of RCC. EVs are lipid membrane-bound vesicles in nanometer size and secreted by almost all cell types into the extracellular milieu. A myriad of bioactive molecules such as RNA, DNA, protein, and lipid are able to be delivered via EVs for the intercellular communication. Hence, the abundant content of EVs is appealing reservoir for biomarker identification through computational analysis and experimental validation. EVs with excellent biocompatibility and biodistribution are natural platforms that can be engineered to offer achievable drug delivery strategies for RCC therapies. Moreover, the multifaceted roles of EVs in RCC progression also provide substantial targets and facilitate EVs-based drug discovery, which will be accelerated by using artificial intelligence approaches. In this review, we summarized the vital roles of EVs in occurrence, metastasis, immune evasion, and drug resistance of RCC. Furthermore, we also recapitulated and prospected the EVs-based potential applications in RCC, including biomarker identification, drug vehicle development as well as drug target discovery.

## Introduction

Renal cell carcinoma, or RCC for short, is one of the most common type of urological cancers that represents ~90% of all kidney malignancies (1). According to updated data provided by the World Health Organization, over 400,000 people were diagnosed with kidney cancer worldwide in 2018, accounting for nearly 3% of all cancers (2). It has been estimated that there will be about 74,000 new cases and 15,000 deaths associated with kidney cancer in the United States in 2020 (3). The 5-year survival rate among RCC patients increased for decades due to the improvement of early-detection techniques and targeted-therapies. The current overall 5-year survival rate of RCC is 75%, decreasing to 70% among patients with regional metastases and 12% among patients with distant metastases (4). Still around one-third of patients diagnosed with RCC had metastases (5). The most common metastatic sites of RCC are lungs, bone, brain, lymph node, and liver might also be involved (6). Surgery is the mainstay curative treatment for localized RCC (7). However, around 40% RCC patients will suffer tumor recurrence after curative surgical resection (8). For patients who present with metastatic RCC or relapses after local therapy, typically require systemic treatment. The current landscape of systemic therapies are consist of small molecule kinase inhibitors, cytokines, and monoclonal antibodies, including checkpoint inhibitors, which have been tested as first-line or second-line therapies (9).

Extracellular vesicles (EVs) are nanometer sized vesicles composed of a lipid bilayer membrane packaging a wealth of bioactive molecules such as RNA, DNA, protein, and lipid. Currently, EVs can be broadly divided into two main types based on the mechanism of biogenesis: one is exosomes which originate from the endosomal system and another one is microvesicles that directly shed from the plasma membrane (10). As Thery et al. mentioned in a review, both exosomes and microvesicles may be co-isolated due to the overlapping characteristics between these two forms of EVs and the limitations of current isolation methods. Therefore, the term exosomes is generally used in literatures to designate a mixed population of EVs without adequate characterization of the intracellular origin (11). Hereafter, we chose to use the generic term “EVs” in this review independent of the term used in the original articles.

With the nanoscale size and double-layered lipid membrane appropriately protecting the cargoes from degradation, EVs stably exist in blood, urine, saliva, and many other kinds of biological fluids. Accumulating evidences indicate that EVs traffic between donor and recipient cells are fundamental phenomenon of the intercellular information exchange, especially in tumor microenvironment (TME). EVs within TME are emerging as crucial contributor to carcinogenesis, angiogenesis, premetastatic niche (PMN) formation, dysfunction of immune system and the dissemination of anti-cancer drugs resistance, adding novel dimension to the complexity of TME (12). Thus, the contents of tumor-derived EVs may be applied as abundant sources to biomarker discovery identified by experimental and computational methods. In addition, EVs with naturally excellent biocompatibility and biodistribution are ideal materials to be exploited or engineered which may offer us achievable drug delivery strategies for cancer therapies (13). Furthermore, it is increasingly clear that mechanisms of EVs biogenesis, secretion and uptake could also provide promising targets for cancer therapy (14).

The past decades have witnessed unprecedented research progresses of EVs, especially for the roles of EVs in different malignant tumors. Nevertheless, to the best of our knowledge, few researchers paid close attention to the roles of EVs in urological malignancies, especially for RCC (15–22). There is still no comprehensive summary highlighting the EVs-based potential applications in RCC either. Hence, this review serves to introduce the latest research progresses in the burgeoning field of EVs, recapitulate the multifaceted functions of EVs in RCC progression. Accordingly, we will also give a perspective of the potential applications of EVs in RCC identified by both experimental and computational methods.

## Biological Features of EVs and Research Techniques

### Biogenesis, Secretion and Uptake of EVs

**Figure 1:**
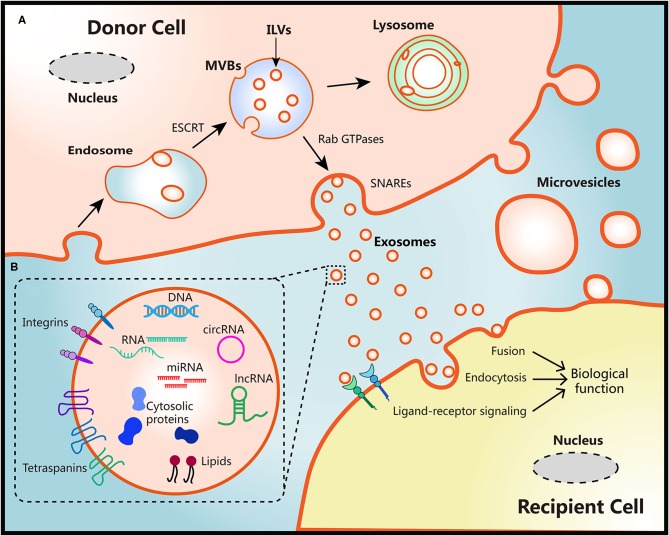
Schematic diagram of the biological features of EVs. **(A)** Biogenesis, secretion and uptake of EVs. During the process of early endosome mature into MVBs, the endosomal membrane invaginate ILVs in the lumen of donor cells, which mediated by the ESCRT machinery. MVB fuse with cell surface and release ILVs as exosomes or degrade in lysosomes. Protein members of Rab GTPases, SNAREs, and synaptotagmin family play vital roles in MVBs trafficking and exosomes secretion. Microvesicles originate from the plasma membrane of donor cells directly. There are three ways to uptake EVs and induce biological functions in recipient cells: fusion with membrane of recipient cells directly, internalization by endocytosis, or activation of ligand-receptor signaling. **(B)** Representative structure and composition of EVs. EVs are nanometer sized vesicles composed of a lipid bilayer membrane. Size of exosomes range from 30 to 150 nm, Diameter of microvesicles range from 50 to 1,000 nm but can up to 10 μm in the case of oncosomes. EVs package various bioactive molecules such as RNA, DNA, proteins, and lipids. Transmembrane including integrins and tetraspanins are also contained in EVs.

The biogenesis of two EVs subtypes are different as shown in Figure 1. Diameter of microvesicles range from 50 to 1,000 nm but can up to 10 μm in the case of oncosomes, which refers to cancer cells-derived microvesicles that contain oncogenic molecules (10, 23). Microvesicles are generated through the direct budding and fission of the cytoplasmic membrane then released into the extracellular space (24). Exosomes originate from multivesicular bodies (MVBs) within endosomal system, ranging from 30 to 150 nm. The endosomal membrane invaginate intraluminal vesicles (ILVs) in the lumen during the mature process of early endosomes into late endosomes or MVBs. The endosomal sorting complex required for transport (ESCRT) machinery plays critical role in this process (10, 25). Moreover, members of the Rab GTPases family, including Rab27a/b, Rab11, and Rab35, are essential coordinators for MVBs trafficking and exosomes secretion (26, 27). The last step of secretion requires the fusion of MVBs with plasma membrane. This process primarily is mediated by soluble *N-*ethylmaleimide-sensitive factor attachment protein receptors (SNAREs) and synaptotagmin family members to release ILVs as exosomes (28). Several studies have also found that Ca^2+^ may be involved in the activation of SNAREs (29, 30).

Once secreted into the extracellular milieu and absorbed by recipient cells, EVs cargoes can be transmitted to recipient cells to induce functional responses and confer new properties then result in phenotypic changes (10). This EVs-mediated interaction requires docking at the plasma membrane of recipient cells via several mediators such as clathrin, tetraspanins, and integrins to activate surface receptors and signaling pathways, being followed by vesicle endocytosis or membrane fusion of recipient cells (10, 31–33). The secretion processes of EVs are evolutionarily conserved among eukaryotes, bacteria, and archaea, which lay the foundation for interspecies transfer of genetic molecules via EVs (34). However, the whole process of exosomes biogenesis and secretion may be influenced by the heterogeneity of donor and recipient cells, different physiological or pathological conditions, making the detailed mechanisms remains elusive (35, 36).

### EVs Composition

Diverse bioactive molecules such as RNA, DNA, proteins, and lipids can be packaged into EVs and secreted out of cell membrane at both local regional and systemic levels (37). A “routine passenger” of EVs is RNA. Both mRNA and microRNA (miRNA) could be loaded and transported through EVs then functioned in recipient cells (38–40). Besides, numerous long non-coding RNA (lncRNA) could also be transferred via EVs, inducing signals and phenotypes changes in a variety of cells in TME (41, 42). Furthermore, more than 1,000 circular RNA (circRNA) were identified in EVs derived from human serum. Interestingly, several circRNAs were highly enriched in EVs compared to the donor cells, which may provide more achievable applications in biomarker discovery (43, 44). Other RNA species were also detected in EVs by RNA deep sequencing analysis, including transfer RNA, ribosomal RNA and piwi-interacting RNA (44, 45).

The presence of DNA within EVs also provide novel insights into the cellular homeostasis and open another intriguing mode of intracellular communication (46). It has been reported that EVs secretion removed various length of chromosomal DNA fragments which were harmful to normal human cells (47). Moreover, studies demonstrated that retrotransposon elements, oncogene amplifications, and other functional DNA fragments that reflected the genetic status of the parent tumor cells were found in EVs (48, 49). Notably, these transposable elements could be encapsulated and transferred from tumor cells to normal cells (50). Thereby it can be inferred that tumor-derived EVs may function as novel mediators of horizontal gene transfer and make contribution to tumor evolution in local or systematical level (51).

As a consequence of the biogenesis, EVs derived from different cell types contain substantial cytosolic proteins, such as Rab GTPase, SNAREs, and Annexins (52). Tetraspanins is a highly conserved family of transmembrane proteins which have been found in EVs from diverse cell types. It is believed that tetraspanins interact or coordinate with other proteins and involve in membrane compartmentalization (53). Members of this family, including CD9, CD63, and CD81, consist part of the most abundant proteins in EVs, thus commonly be used as protein markers for EVs characterization (54). In addition, increasing evidences have demonstrated the presence of several transporters and enzymes in EVs with full activity (55–57). Thus, it can be inferred that the change of EVs components can be connected with the *in vivo* fate of drugs.

### EVs Isolation and Characterization

Since research field of EVs has achieved high-speed development in the past few decades, many techniques have been used to isolate and characterize EVs. At present, the frequently used techniques for EVs isolation can be summarized into five broad categories: differential ultracentrifugation (UC), polymer-based precipitation, particle size-based techniques, immunological capture, and microfluidic techniques (58). As one of the most traditionally and widely used method, differential UC is suitable for most sources of EVs, even though it is laborious, time-consuming, and inaccessible. Several commercial isolation kits are developed based on above theories and techniques to isolate EVs more efficiently and precisely. However, according to results of a recent benchmark study, a large quantity of non-vesicular contaminants may be co-isolated by these kits. While the purity of EVs isolated by differential UC was much higher than commercial kits (59). More recently, microfluidic-based platforms have generated heightened interest. Based on specific capture of the surface marker or the specific size and density of EVs subsets, microfluidic-based platform can provide advantages such as low consumption, ready portability, with high throughput, and high precision (60). Since there is still no consensus on a “gold standard” method for EVs isolation and purification, comparison study is still needed to analyze the parameters of EVs isolated by different methods. According to a global survey in 2015 conducted by the International Society for Extracellular Vesicles (ISEV), around 81% of respondents chose differential UC as their primary isolation method, around 59% of respondents used a combination methods of differential UC with other techniques (61). In terms of EVs characterization, multiple techniques based on biophysics and molecular biology have been developed and applied. Three of the most common methods are western blotting for identification of specific protein marker, electron microscopy for detection of structural information and nanoparticle tracking analysis for quantification of EVs size and concentration, respectively. Generally speaking, two or more complementary methods are necessary to assess the results of separation methods as ISEV recommended (62).

## Roles of EVs in RCC

**Figure 2:**
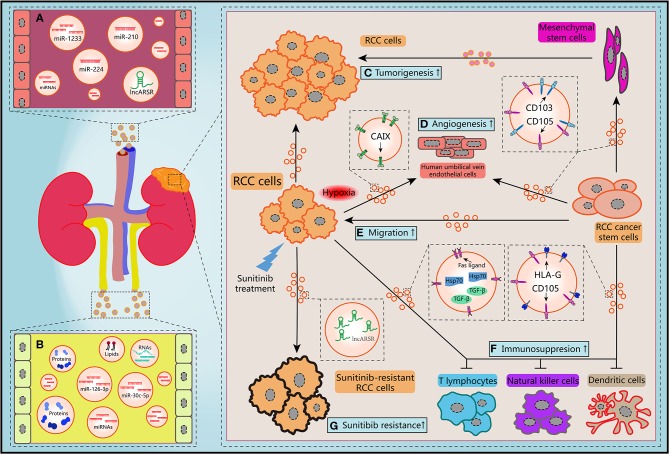
Schematic diagram of the biological features of EVs. **(A)** Circulating EVs in blood contain potential biomarkers of RCC. **(B)** Circulating EVs in urine contain potential biomarkers of RCC. **(C)** RCC-derived EVs and mesenchymal stem cells-derived EVs promoted the tumorigenesis of RCC cells. **(D,E)** Migration ability of RCC cells and angiogenesis of human umbilical vein endothelial cells and could be improved by hypoxic RCC cells released EVs containing CAIX, CD103-positive or CD105-positive RCC CSCs-derived EVs. **(F)** RCC cells-derived EVs and RCC CSCs-derived EVs facilitated the immunosuppression of immune cells. **(G)** Sunitinib treatment induced RCC cells secreted EVs delivering lncARSR to increase the drug resistance of RCC cells.

EVs is employed by tumor cells to deliver bioactive molecules directing to not only tumor cells but also tumor-associated cells including fibroblasts, endothelial cells, immune cells, and cancer stem cells (CSCs) (63, 64). Reciprocally, EVs derived from non-tumor cells also have influence on tumor progression in TME. Therefore, these multidirectional communications via EVs make TME becoming a more complex network, which draw accumulating attention of researchers in recent years. Herein we reviewed the latest studies about roles of EVs in carcinogenesis, cancer metastasis, immune evasion, and drug resistance of RCC (Figure 2).

### Tumorigenesis

EVs secreted by different cells in TME may make contributions to RCC progression and development. Jiang et al. revealed that EVs secreted by RCC cell line OS-RC-2 could inhibit hepaCAM expression, a tumor suppressor frequently lost in various types of human cancers, and promote cell proliferation in a p-AKT-dependent pathway (65). By use of cell culture and nude mice xenograft model, Du et al. claimed that EVs released by human Wharton's jelly mesenchymal stem cells induced HGF expression, activated AKT and ERK1/2 signaling pathways, then promoted the proliferation and aggressiveness of RCC cells both *in vitro* and *in vivo* (66). By using next-generation sequencing, Song et al. found the levels of EVs-contained miR-30c-5p in RCC cell lines 786-O and ACHN were significant lower than that in human renal proximal tubular cell line HK-2. Consistently, the expression pattern of miR-30c-5p was significant different in urinary EVs from healthy controls and patients of clear cell RCC (ccRCC), which is the predominant RCC type. Heat-shock protein 5 was identified as a direct target of miR-30c-5p. Gain-of-function study showed that overexpression of miR-30c-5p inhibited ccRCC progression both *in vitro* and *in vivo* (67). Considered together, these data suggest that EVs may transfer various cargoes between heterogeneous cells within TME, initiate the critical regulation of the tumorigenesis to support the growth of RCC cells.

Hypoxia is one of the distinguishing features of TME in many solid tumors including RCC. Carbonic anhydrase IX (CAIX), a cellular hypoxia biomarker that overexpress in RCC with *von Hippel-Lindau* (*VHL*) gene mutation, is involved in proliferation and transformation of RCC cells (68). It has been revealed that abundant CAIX proteins were detected in EVs released from RCC cell lines. Result of *in vitro* angiogenesis assays demonstrated that hypoxic RCC cells could release EVs containing CAIX and promote the migration and tube formation abilities of human umbilical vein endothelial cells (69). Several researchers have also provided direct evidences that hypoxia not only regulated the tumorigenic potential of epithelial cells, but also contributed to EVs production of tumor cells in response to low pH and oxidative stress (70, 71). Wang et al. reported that acute hypoxia condition induced by CoCl_2_ treatment upregulated miR-210 expression in EVs which derived from both normal renal cells and RCC cells, especially for metastatic RCC cell line (72). Interestingly, EVs secreted by hypoxic cells are more easily absorbed by hypoxic cells (73). Hitherto, there is limited knowledge about the mechanism of how hypoxia orchestrate the biogenesis and secretion of EVs. Nevertheless, it can be concluded that hypoxia-induced EVs derived from stromal and tumor cells are crucial mediator in the process of tumorigenesis and TME rebuilding.

### Tumor Metastasis

Recent years, numerous investigations have revealed the significant influence of EVs on both regional and distant metastatic processes, including coagulation, vascular leakiness, reprogram of stromal recipient cells, and formation of PMN (74). However, the roles of EVs in RCC metastasis are still need to be unraveled. It has been shown that MMP-9 and CXCR-4 are closely associate with tumor metastasis and highly express in different cancer types. Chen et al. revealed that expression levels of these two proteins were upregulated after co-cultured RCC cell line 786-O with EVs shed from itself, which resulted in the improvements of the migration and invasion abilities and suppression of the adhesion ability (75). Camussi's team identified a subset of tumor-initiating cells expressing mesenchymal stem cell marker CD105 from human RCC specimens in a previous work. They found that EVs released by renal CD105^+^ CSCs could trigger angiogenesis both *in vitro* and *in vivo*, and enhanced the lung metastases induced by injection of renal tumor cells intravenously. Furthermore, mRNAs and miRNAs implicating in tumor progression and metastasis were identified through molecular characterization of EVs (76). Subsequently, Camussi et al. reported that renal CSCs-derived EVs could stimulate persistent phenotypical changes in mesenchymal stem cells *in vitro* and support the tumor growth and vascularization when co-injected with RCC cells *in vivo* (77). Their conclusions unveiled that EVs shed from a subtype of renal CSCs may play critical roles in the TME modification, PMN formation, and metastasis of RCC in lung, which is one of the most common site of RCC metastasis.

Recently, Wang et al. demonstrated that CD103^+^ CSCs, another subtype of renal CSCs, could release EVs enwrapping miR-19b-3p and deliver to RCC cells to initiate epithelial-mesenchymal transition (EMT) via suppressing the expression of PTEN. Quantitative detection of expression changes of EMT markers such as N-cadherin, Vimentin and Twist showed that CD103^+^ CSCs EVs derived from RCC patients with lung metastasis presented significant effects on EMT. Notably, results of flow cytometry quantification also showed that the ratio of CD103^+^ EVs over total EVs was higher in blood samples of RCC patients with lung metastasis than non-metastasis patients (78). Therefore, it can be inferred that EVs-contained CD103 may be involved in the organotropism of RCC. Additionally, previous work suggested that tetraspanins and integrins were also associated with metastasis organotropism (79, 80). Typically, integrins α_6_ and α_v_ were closely relevant to lung and liver metastases, respectively (33). Since lung and liver are common sites for RCC metastasis, we can believe that integrins α_6_ and α_v_ may present in RCC-derived EVs and address these EVs to specific organs. Hence these endogenous surface molecules of EVs provide us crucial clues to understand the complex mechanism of tumor metastasis. It will be promising to develop indicators of metastatic prognosis and selective target-binding therapeutics for RCC treatment through unraveling the functions these transmembrane proteins.

### Immune Evasion

In the past decade, the deep comprehension of communication between the immune cells and malignant tumor cells in TME has become a popular research field. Emerging investigations advocated that EVs are active players in this scenario (81). However, this interaction can be hijacked by tumor cells to facilitate immune evasion and stick many anti-cancer therapeutic strategies. Studies showed that the activation of T cells and the differentiation processes of monocytes to dendritic cells (DC cells) were both impaired by EVs derived from renal CD105^+^ CSCs (82). This immune inhibitory effect was mediated by HLA-G, an antigen highly overexpressing in RCC and facilitating to immunosuppression (83). HLA-G blockade markedly relieved the inhibitory effect of EVs on DC cells differentiation. It has also been verified that EVs purified from RCC cell line ACHN contained Fas ligand and contributed to apoptosis of Jurkat T lymphocyte and immune evasion of RCC cells. These effects could be rescued by soluble Fas treatment (84). Natural killer (NK) cells are crucial player in the innate immune system, possessing strong abilities to control and kill tumor cells. Xia et al. found that EVs derived from primary RCC cells contained TGF-β, a major immunosuppressive cytokine. Co-culturing these EVs with NK cells exacerbated the dysfunctions of NK cells in a TGF-β/SMAD-dependent manner (85). Furthermore, Diao et al. elucidated that Hsp70 protein was more enriched in EVs than that in whole-cell lysates of Renca cells which is a cancer cell line of murine kidney. EVs-contained Hsp70 triggered the phosphorylation of Stat3 through regulating TLR2-MyD88 pathway and impeding the activity of the myeloid-derived suppressor cells (86). Considered together, these conclusions suggest that RCC cells may secrete EVs to interfere the immune system and support evasion of innate immune surveillance. Potential drug targets or biomarkers of the immunotherapy can be developed by clarifying the detailed mechanism of intercellular communication between cancer cells and immune cells.

Immunotherapy is one of the most promising therapeutic approach in multiple cancer types including RCC. Immune checkpoint protein inhibitors, especially antibodies against programmed cell death-1 (PD-1) and its ligand programmed death-ligand 1 (PD-L1), have elicited anti-cancer effects and long-lasting alleviation in melanoma, lymphoma, bladder cancer, non-small-cell lung cancer, RCC, and many other malignancies (87). However, only limited subset of patients exhibited durable response to immunotherapies. The total respond rate of anti-PD-1/PD-L1 therapy is merely around 10–30% (88). Previous studies have identified EVs-contained PD-L1 in diverse sources, including plasma of head and neck cancer glioblastoma, and melanoma patients as well as culture medium of breast cancer cell lines (89–94). A recent work demonstrated that EVs could support tumor growth by carrying PD-L1 and suppressing T cell activation in draining lymph nodes. Genetic blockade of EVs-contained PD-L1 induced long-term and systemic anti-tumor effects (95). Most recently, several novel methods were developed to quantitate the PD-L1 level in EVs. These newly approaches were higher in sensitivity, time-saving, and easily operated compared with ELISA-based canonical methods (96, 97). However, to the best of our knowledge, yet still no research focus on the PD-L1 in RCC-derived EVs. Above findings enlighten us that inhibition of EVs-contained PD-L1 may be an alternative therapy for RCC treatment, especially for RCC patients that are resistant to anti-PD-L1 antibodies. Meanwhile, EVs-carried molecules represented by PD-L1 may serve as reliable biomarkers for immunotherapies.

### Drug Resistance

Accumulating evidences corroborate that EVs make non-negligible contributions to the resistance of anti-cancer drugs. The horizontally intercellular transmit of drug resistance are mediated by EVs cargoes including drug-efflux transporters, miRNAs, lncRNAs (98). Corcoran et al. established and characterized docetaxel-resistant variants of two prostate cancer cell lines by a serial assays including cross-resistance, morphology, multi-category phenotypes, and EVs secretion. They revealed that EVs released from docetaxel-resistant prostate cancer cells subverted sensitive cells to docetaxel-resistant phenotype through the involvement of EVs delivering multidrug resistance protein 1. Consistent results were presented when co-cultured docetaxel-sensitive prostate cancer cells with serum-derived EVs from prostate cancer patients before and after commencing docetaxel treatment (99). As a vital organ for the elimination and reabsorption of therapeutic drugs, kidney contain various drug transporters in proximal tubules. Thereby the variability of renal drug transporters will impact the processes of drug disposition (100). However, there is still no study focus on the drug resistance in RCC mediated by EVs-contained drug transporters.

Since several receptor tyrosine kinases relevant to angiogenesis and homeostasis of TME are overexpressed predominantly due to inactivation of *VHL* gene in ccRCC, inhibitors targeted receptor tyrosine kinases such as sunitinib have become the one of first-line therapies for RCC treatment (101). However, the clinical benefit of sunitinib treatment in ccRCC patients is limited due to inherent or acquired resistance. As such, the biological basis for resistance to sunitinib therapy and the clinical approach in this setting is of heightened interest of investigators (102). Qu et al. obtained sunitinib-resistant RCC cells through cycles of sunitinib treatment to nude mice with serial xenografts. Then lncRNA required for sunitinib resistance in RCC was identified by three rounds of screening sequentially. Firstly, lncRNA expression profiles between parental and sunitinib-resistant RCC cells was compared by lncRNA microarray. Then they established patient-derived xenograft models of RCC and mimic sunitinib therapy. Eight lncRNA candidates were consequently selected to loss-of-function analysis by RNAi in sunitinib-resistant RCC cells. LncARSR was eventually identified as a highly abundant lncRNA in sunitinib-resistant ccRCC cells, which could favor sunitinib resistance via competitively binding both miR-34 and miR-449 to improve AXL and c-MET expression. More interestingly, lncARSR could be secreted and delivered via EVs to transform the phenotype of recipient cells from sunitinib-sensitive to sunitinib-resistant and lead to the dissemination of sunitinib resistance (103). Overall, it is valuable to clarify the various mechanisms of anti-cancer drugs resistance mediated by EVs, which may further help us to identify desirable biomarkers that can be used in drug response and identify novel targets to restore therapeutic approaches.

## Clinical Implications of EVs in RCC

Recent years, many reviews have summarized the clinical implications of EVs in a variety of cancer types. Since the composition of the original cells can be reflected in the cargoes of EVs in a real-time mode, the initial interest of clinical implications is to find vital biomarkers from this favorable reservoir. EVs are natural nanoscale vesicles as ideal engineering platform owing to their unique advantages such as low toxicity and long-term stability in biofluids (104). EVs-based drug target discovery is also draw considerable attention of researchers due to recent findings. Moreover, RCC is still a malignant tumor with unpredictable progression, limited effective therapies and poor clinical prognosis. The progresses of clinical application of EVs in RCC is also relatively lag behind than that in other cancer types. Accordingly, the demonstrated and conceivable clinical implications of EVs in RCC will be discussed here from following aspects.

### EVs-Derived Biomarkers for RCC

Owing to the encapsulation by vesicle membrane, the bioactive molecules within EVs are free from degradation by exogenous nucleases or proteases and stable in biological fluids (15). These abundant content which may be reliable biomarkers for prediction of RCC progression have been extensively investigated. Previously, Zhao et al. reported that the expression level of miR-210 was differentially higher in primary RCC tissues of 32 patients than non-tumor renal parenchymas. Results of receiver operating characteristic (ROC) analysis also showed that ccRCC patients and healthy individuals could be discriminated by the average level of cell-free miR-210 in serum (105). They assessed expression levels of three miRNAs (miR-210, miR-1233, and miR-15a) in serum-derived EVs in a follow-up work. Results of ROC analysis showed that it was feasible to use miR-210 and miR-1233 but not miR-15a as diagnostic biomarkers (106). Consistently, a recent study confirmed the expression level of miR-210 in serum-derived EVs was significantly higher in RCC patients than healthy controls (72). Similarly, expression level of miR-224 was also overexpressed in cancer tissues of ccRCC patients (107, 108). The level of serum EVs-contained miR-224 was significantly correlated with progression-free survival (PFS) or overall survival (OS) of ccRCC patients (109). Moreover, a study evaluated the possibility of miRNAs from plasma-derived EVs for RCC prognosis by RNA sequencing. Results of Kaplan-Meier analysis confirmed the correlations of three miRNAs with OS of RCC patients, including miR-let-7i-5p, miR-26a-1-3p, and miR-615-3p (110).

Urine as a dynamic biofluid is also a promising source for RCC biomarker development rather than a waste product of body. Urinary EVs can be released from every renal epithelial cell type facing the urinary tract. Therefore, the cargoes of urinary EVs may be accessibly real-time signals for renal dysfunction. However, only few researchers attempted to find bioactive molecules from urinary EVs and these snapshots need to be further characterized (18). Study reported that combinations of urinary EVs-derived miRNAs (miR-449a, miR-34b-5p, or miR-486-5p with miR-126-3p) had the power to distinguish healthy controls, patients with benign renal tumors, and patients with early-stage or advanced ccRCC (111). It has also claimed that the level of miR-30c-5p within the urinary EVs was significantly decreased in ccRCC patients but not in other urological malignancies samples (67). In addition, differential levels of miR-150 and miR-205 were found in EVs isolated from 786-O and HK-2 cell lines (112). Our previous work showed that the lost expression of organic cation transporter 2 were partly due to the downregulation by miR-489-3p and miR-630. Interestingly, miR-489-3p and miR-630 were more abundant in EVs than donor cells (113, 114). Therefore, these findings of fundamental work may also have translational value to provide clues for RCC biomarker discovery in a certain extent.

In addition to miRNAs, other content of EVs also have potential to be developed as biomarkers for RCC. As mentioned above, lncARSR was elucidated as a mediator of the transmission of sunitinib resistance, which could be enwrapped and delivered through EVs. Qu et al. further revealed that circulating lncARSR could be utilized as indicator to predict sunitinib response in RCC patients (103). Moreover, Palma et al. reported that the mRNA levels of *GSTA1, CEBPA*, and *PCBD1* genes in urinary EVs were lower in RCC patients than that in control subjects and this pattern backed to normal level after 1 month of nephrectomy (115). In 2012, Boccio et al. established a hyphenated micro LC-Q-TOF-MS platform to profile the lipid repertoire of human urinary EVs. A comparative analysis for lipid content in urinary EVs purified from RCC patients and healthy subjects was performed for the first time (116). Similarly, a proteomics study in 2013 reported that the protein composition of urinary EVs was substantially different in RCC patients and control subjects. Results presented for the first time that considerable number of proteins were significantly enriched in RCC patients, including Ceruloplasmin, Podocalyxin, Dickkopf related protein 4, MMP9 and CAIX (117). A recent work reported that Azurocidin was highly enriched in EVs isolated from tumor tissues of ccRCC patients than adjacent normal tissues. Importantly, Azurocidin content was also significantly higher in serum EVs from ccRCC patients compared to healthy controls (118). These tentative work provided valuable indications for exploiting potential mRNA, lipid, and protein biomarker for RCC from urinary EVs. Taken together, it can be concluded that multiple EVs cargoes derived from different kinds of biofluids are promising non-invasive biomarkers for early diagnosis and treatment of RCC. The potential biomarkers derived from EVs which have been validated in clinical samples of RCC are listed in Table 1.

**Table 1:** EVs derived potential biomarkers with clinical significance for RCC.

**Type**	**EVs source**	**EVs cargoes**	**Analysis method**	**Cohorts**	**Clinical significance**	**Year**	**References**
Lipid	Urine	LysoPE etc. 196 differential signals	microLC-Q-TOF-MS	8 ccRCC patients, 8 HS	48 differential lipidomes (22 upregulated and 26 downregulated in RCC)	2012	(116)
lncRNA	Plasma	Circulating lncARSR	qRT-PCR	71 advanced ccRCC patients, 32 HS	Differentiated ccRCC patients from healthy controls; High lncARSR levels in pre-therapy correlated with PFS independent of clinical characteristics	2016	(103)
mRNA	Urine	GSTA1, CEBPA, PCBD1	Microarray, qRT-PCR	46 RCC patients (33 with ccRCC), 22 HS	Significant lower in ccRCC patients than HS and increased to normal level 1 month after nephrectomy	2016	(115)
miRNA	Plasma	miR-let-7i-5p, miR-26a-1-3p, miR-615-3p	RNA-sequencing, qRT-PCR	44 and 65 metastatic RCC patients for screening and validate cohort, respectively	Low levels correlated with poor OS of mRCC patients, independent of age, gender, tumor grade, stage at diagnosis, coagulative necrosis, or sarcomatoid differentiation	2017	(110)
	Serum	miR-1233, miR-210	qRT-PCR	82 ccRCC patients, 80 HS	Both significant higher in ccRCC patients than HS independent of gender, age, or ccRCC grade	2018	(106)
	Serum	miR-210	Microarray, qRT-PCR	45 pre-operative and 35 post-operative ccRCC patients, 30 HS	Significant higher in ccRCC patients than HS, and in pre-operative than post-operative samples	2019	(72)
	Serum	miR-224	qRT-PCR	108 ccRCC patients	High level correlated with shorter PFS, CSS and OS of ccRCC patients	2017	(109)
	Urine	miR-126-3p	Microarray, qRT-PCR	81 ccRCC patients, 33 HS	Differentiated ccRCC patients from HS	2016	(111)
	Urine	miR-126-3p combined miR-449a	Microarray, qRT-PCR	81 ccRCC patients, 33 HS	Differentiated ccRCC patients from HS		
	Urine	miR-126-3p combined miR-34b-5p	Microarray, qRT-PCR	81 ccRCC patients, 33 HS	Differentiated ccRCC and small renal masses (pT1a, ≤ 4 cm) patients from HS, respectively		
	Urine	miR-126-3p combined miR-486-5p	Microarray, qRT-PCR	24 benign renal tumor patients, 33 HS	Differentiated benign patients from HS		
	Urine	miR-30c-5p	RNA-sequencing, qRT-PCR	70 early-stage ccRCC patients, 30 HS	Significant lower in early-stage ccRCC patients than HS	2019	(67)
Protein	Urine	Matrix metalloproteinase 9, Ceruloplasmin, Podocalyxin, Dickkopf related protein 4, Carbonic anhydrase IX	LC-MS/MS, western blotting	9 ccRCC patients, 9 HS	Significant higher in ccRCC patients than HS	2013	(117)
	Urine	Aquaporin-1, Extracellular matrix metalloproteinase inducer, Neprilysin, Dipeptidase 1, Syntenin-1	LC-MS/MS, western blotting	9 ccRCC patients, 9 HS	Significant lower in ccRCC patients than HS		
	Serum	CD103	Flow cytometry	76 and 133 metastatic or non-metastatic ccRCC patients, respectively	Higher ratio of CD103^+^ EVs over total EVs in samples of metastatic patients than non-metastatic patients	2019	(78)
	Serum	Azurocidin	LC-MS/MS	19 ccRCC patients, 10 HS	Significant higher in ccRCC patients than HS	2018	(118)
	Tissue	Azurocidin	LC-MS/MS	20 paired tumor and adjacent normal tissues of ccRCC patients	Significant higher in ccRCC patients than HS		

### EVs-Based Drug Vehicles and Targets in RCC

The biological characteristics make EVs can be harnessed as vehicles for therapeutic agents to improve curative effect. Numerous clinical and preclinical trials have suggested that these EVs-based drug vehicles and therapies are promising, feasible and well-tolerated (119–121). There are two basic approaches to load cargoes into EVs: exogenous loading and endogenous loading. Exogenous modification can be achieved after collection of EVs, with encapsulation of small molecules, proteins, and RNAs into or onto EVs via diverse methods including co-incubation, electroporation, and sonication (121). Tian et al., developed a tumor-targeting EVs from mouse immature DC cells expressing a well-characterized EVs membrane protein (Lamp2b) fused to integrin α_v_-specific iRGD, which is a new tumor-homing and penetrating peptide. After loaded with doxorubicin via electroporation, this delivery platform showed high efficiency in tumor-targeting and doxorubicin delivery to integrin α_v_-positive breast cancer cells both *in vitro* and *in vivo* (122). Wan et al. developed a nucleolin-targeting aptamer AS1411 which covalently conjugated to cholesterol-PEG and anchored onto membrane of mouse DC cells. Subsequently, EVs were obtained from this modified DC cell model and loaded with paclitaxel by sonication. Results of cancer treatment in xenograft nude mice showed that engineered EVs enhanced therapeutic efficacy with low systemic toxicity (123). We can believe that along with the detailed mechanism of EVs-mediated metastasis organotropism are being clarified, EVs are promising material to achieve drug-targeting delivery for cancer treatment. However, the immune responses are need to be considered seriously. Additionally, the production yield is also a challenge for applying engineered EVs in tumor-targeting delivery.

Alternatively, cargo of EVs can be endogenously loaded through genetically manipulating the donor cells to overexpress bioactive molecules and employed as EVs-based vaccines or imaging tools. With significant higher level in surface of RCC cells than normal renal cells, RCC-associated antigen G250 could be served as one of the therapeutic targets (124). EVs containing G250 or other RCC-specific antigens may be novel approaches to develop EVs-based cancer vaccines for RCC treatment. It has been shown that modified RCC cells released EVs expressing both glycolipid-anchored-IL-12 and G250, which efficiently promoted the proliferation of antigen-specific cytotoxic T lymphocytes and enhanced cytotoxic effects (125). Notably, there is a risk of mixing pathogens such as viruses with EVs since these nanometric vesicles have similar biophysical properties (126). Hence a standard operating procedure is very necessary when isolate EVs as cancer vaccine. By combining a Cre recombinase-based system with high-resolution fluorescence imaging techniques, Zomer et al. realized the visualization of intracellular EVs exchange within local and distant tumor sites *in vivo*. Results showed that less malignant tumor cells presented heightened migratory ability after taken up the EVs released by highly malignant tumor cells (127). Moreover, several other molecular imaging strategies have also been utilized to monitor and determine the biodistribution of EVs *in vivo*, including bioluminescence, nuclear, and magnetic resonance imaging techniques (128). These interesting findings and advanced techniques make it clear that EVs-based modification can be used to achieve the phenocopying of tumor cells and visualize cancer development process *in vivo* in the future.

Drugs targeting vital steps in formation, release or uptake of EVs may also be served as effective adjuvants for cancer treatment. Datta et al. utilized quantitative high throughput screen assay to find active compounds targeting the formation and release of EVs in prostate cancer cells. Totally five and six lead compounds were validated as potent inhibitors and activators, respectively (129). In another review, two groups of candidate drugs were broadly classified according to the mechanisms of modulating EVs biogenesis or secretion. One is compounds that specifically inhibit EVs trafficking, including calpeptin, manumycin A, and Y27632. Another group is compounds that specifically disrupt lipid metabolism, including pantethine, imipramine, and GW4869 (130). Interestingly, Ortiz et al. identified that reserpine, a commonly used anti-hypertensive drug since 1955, could alter the fusion process of lipid membrane and then inhibit PMN formation that was induced by melanoma-derived EVs. Their findings indicated that tumor-derived EVs could “educate” healthy cells to facilitate tumor metastasis. Meanwhile agents like reserpine can interfere this education process and play a defensive role on EVs uptake. Thus, it is valuable to repurpose these drugs as adjuvant treatment for metastatic cancer therapy (131). More recently, sulfisoxazole, an oral antibacterial drug approved by US FDA, was screened out as inhibitor of EVs secretion in breast cancer cells. Through targeting endothelin receptor A, sulfisoxazole promoted the degradation of ESCRT-dependent MVB, suppressed biogenesis and secretion of EVs, as well as significantly inhibited the growth and metastasis of breast cancer cells without notable toxicity (132). These important findings enlighten us drug repurposing can be harnessed as approaches to block EVs functions in tumor progression.

### Potential Application of Artificial Intelligence in EVs Research

Artificial intelligence (AI) refers to the simulation of human intelligence in machines. AI approaches have the potential to enhance the qualitative interpretation of cancer imaging by expert clinicians in three main tasks: computer-aided detection of tumor sites, characterization of intra-tumor heterogeneity and variation, as well as temporal monitoring of tumor changes (133). As a specific subset of AI approaches, machine learning (ML) are able to interpret complex data and leverage the detailed information to make accurate prediction or decision. Studies have demonstrated that deep learning frameworks can be applied to distinguish major subtypes of RCC using histological or computed tomography images (134, 135). Similarly, ML algorithms also have the power to analyze a substantial amount of images that are produced by EVs purification and characterization processes. Studies showed that these biophysical parameters of EVs could be assessed by ML algorithms to identify the subpopulation of EVs or even further predict the original donor cells (136, 137). Due to the incredible amount of EVs and the need for downstream analysis during each study, multiparameter results of EVs characterization are particularly amenable to ML algorithms. A preliminary work of Borgovan et al. reported that ML algorithms could distinguish the heterogeneous EVs derived from blood samples with healthy or leukemic phenotypes based on data sets collected from a nanoparticle tracking analysis, thus improved the accurate of EVs classification (138).

At present, one of the most challenges in the field of biomarker discovery is how to decipher the huge amount of garbled information within EVs. AI approaches are becoming trustworthy solutions to this given problem as they are able to modelize complicated network and leverage valuable information within observed data to accurately estimate and predict new samples. Early in 2003, Won et al. had identified five protein biomarkers of serum by using a mass spectrometry-based protein profiling and AI analysis and then successfully differentiated RCC from healthy subjects and other urological diseases (139). Moreover, Zheng et al. developed a novel diagnosis tool to predict early-stage RCC patients which depended on a biomarker cluster that was identified by serum metabolomics method and ML algorithms (140). Meanwhile, unprecedentedly massive data of EVs are also being generated by various “omics” technologies including genomics, transcriptomics, proteomics, metabolomics, glycomics, and lipidomics (141). Several online databases have been established to categorize the RNAs, lipids, proteins, and metabolites within EVs, which have been summarized in Table 2 (142–151). These integrative resources will favor researchers to outline the landscape of EVs in cancer progression and identify relevant biomarkers more quickly and more accurately.

**Table 2:** EVs related online databases.

**Database**	**Publish date**	**Overview**	**Update date**	**References**
EVmiRNA	2019	Comprehensive miRNA expression profiles in 462 EVs small RNA-sequencing datasets from 17 tissues/diseases	2019	(142)
EVpedia	2013	High-throughput datasets of EVs components (proteins, RNAs, and lipids) from prokaryotic and eukaryotic EVs	2013	(143)
EV-TRACK	2017	Experimental parameters of EV-related studies	2019	(144)
ExoCarta	2009	Identified contents (protein, mRNA, miRNA, and lipids) of exosomes in multiple organisms from 286 studies	2016	(145)
exoRBase	2018	Exosomal RNA (circRNA, lncRNA, and mRNA) derived from RNA-sequencing data analyses of human blood	2019	(146)
Exosome Gene Ontology Annotation Initiative	2015	GO annotations of human exosomal proteins	2015	(147)
Plasma Proteome Database	2014	Annotation of 318 identified proteins of EVs from plasma	2014	(148)
Urinary Exosome Protein Database	2004	Mass spectrometry data of 1,160 proteins derived from urinary exosomes isolated from healthy human volunteers	2009	(149, 150)
Vesiclepedia	2012	Compendium of molecular data (lipid, RNA, and protein) identified in different classes of EVs from 1,254 studies	2019	(151)

Integrating EVs-derived biomarkers with ML algorithms to analyze patterns in massive data sources such as gene expression, protein expression, or digital pathology data may obtain a higher diagnostic efficacy of the diagnosis. Chen et al. profiled four surface biomarkers including HER2, GPC-1, EpCAM, and EGFR from serum-derived EVs through DNA points accumulation for imaging in nanoscale topography. They implemented an integrated platform combining EVs identification with quantitative analysis and accurately differentiated pancreatic cancer and breast cancer from unknown samples (152). Additionally, advanced techniques such as microfluidic make it possible to separate EVs on a single chip. In a previous study, Ko et al. developed a multichannel microfluidic platform combining with ML algorithms that specifically isolated EVs from clinical plasma samples, quantitatively detected the RNA profile inside of EVs, and distinguished pancreatic cancer patients with healthy controls (153). They subsequently exploited another workflow that integrated a magnetic capture system with RNA sequencing and ML algorithms. This system purified a subpopulation of EVs and identified a panel of 11 miRNAs from EVs which could classify distinct cancer states in a transgenic mouse model (154). Thus, it is also a feasible strategy to combine upstream isolation methods with downstream ML algorithms to realize the development of “on-a-chip” platform for systemically purification and determination of EVs-derived biomarkers.

Moreover, AI approaches promises to make great strides in almost all stage of drug discovery, including target validation, biomarker identification, and analysis of clinical trial information (155). Since the drug data sets are becoming dynamic, heterogeneous and large scale, state-of-the-art AI approaches such as deep learning and innovative modeling methods provide new answers to efficacy and safety evaluations of drug candidates based on big data modeling and analysis (156). Donner et al. reported a novel method for computational drug repositioning by taking advantage of neural network. They revealed previously unnoticed functional relationships between different compounds based on denoise gene expression data rather than structural similarity (157). Hence AI approaches can build bridges between abundant data sources from high-throughput experiments with gene expression profiles and massive drug candidates. The information of EVs content is also increasingly rich in data. Meanwhile the downstream effects of EVs in cancer progression are non-linear. It is reasonable to assume that the ability of AI to mining valuable information presents new opportunities for novel target identification and validation for EVs-based anti-cancer therapies. Therapeutic Target Database (TTD, http://db.idrblab.org/ttd/) has been established to integrate information of early drug candidates and therapeutic targets that contain expanded knowledge of target regulators such as miRNAs, transcription factors and other interacting proteins (158). Database with molecular information about drugs such as DrugBank (https://www.drugbank.ca/) include comprehensive data of the influence of hundreds drugs on metabolite levels, gene expression levels and protein expression levels, enabling us to find more connections of EVs content changes with drugs (159). Altogether, these important approaches may provide novel research tools to fundamental studies of EVs biology and translational studies of EVs-based therapies. Clearly, more work is need to be deployed in this scenario to figure out the completed mechanisms of EVs biogenesis, secretion and uptake, which may reward us valuable drug targets by using advanced AI approaches.

## Prospects

EVs are attracting increasing attention in cancer research due to its various roles in intracellular communication during cancer progression. However, RCC is relative unnoticed in this research hotspot compared with other cancer types. In this review, we recapitulated the roles and clinical implications of EVs in RCC. Diverse bioactive molecules carried by EVs regulate almost all processes of RCC, such as tumorigenesis, metastasis, immunosuppression, and drug resistance. Due to the unique function of kidney in urinary system, both blood and urine are valuable biofluids with abundant EVs, which are readily accessible sources for biomarkers discovery. Moreover, multiple potential applications can be developed to provide novel strategies for diagnosis and treatment of RCC, including but not limited to EVs-based cancer vaccine, *in vivo* imaging technique, targeted drug delivery system, and drug discovery. But it is noteworthy the detailed mechanisms and effects of EVs on RCC progression are still to be further clarified. The gaps between digital analysis and experimental validation are still need to be solved. Meantime there are still a variety of challenges for the clinical use of EVs in RCC. Standard operating procedure for EVs isolation, quantification, and analysis are still deficiency, especially for biofluids sample. The stability and the unknown side effects of EVs-based therapy must to be considered and assessed. Moreover, High-quality data sets are required in terms of the AI-aided drug target discovery based on EVs. Taken together, extensive work need to be launched to make a better understanding of roles of EVs in RCC progression and make the potential clinical utilities for EVs in RCC therapies come true.

## Author Contributions

ZQ was a major contributor in searching the literature and writing the manuscript. QX and HH reviewed the manuscript and provided significant revisions. LY and SZ gave critical advice and guidance throughout the whole process of this study. All the authors read and approved the final manuscript.

## Conflict of Interest

The authors declare that the research was conducted in the absence of any commercial or financial relationships that could be construed as a potential conflict of interest.
